# Supplementary material for: Hypergravity Increases Blood–Brain Barrier Permeability to Fluorescent Dextran and Antisense Oligonucleotide in Mice
Source: Cells. 2023 Feb 24;12(5):734. doi: 10.3390/cells12050734 (PMC10000817; doi:10.3390/cells12050734)
Supplement: Supplementary file 1 [file cells-12-00734-s001.zip › cells-2211115-supplementary.pdf]

**Table S1: List of genes modulated by 2g**

| Abbreviation | Gene name | Role in BBB | Genecard id |
|---|---|---|---|
| ***Actn1*** | Actinin alpha 1 | Endothelial cell cytoskeleton | GC14M068874 |
| ***Actn2**** | Actinin alpha 2 | Endothelial cell cytoskeleton | GC01P236686 |
| ***Actn4*** | Actinin alpha 4 | Endothelial cell cytoskeleton | GC19P038647 |
| ***Cdh2*** | Cadherin 2 | Adherens junction | GC18M028088 |
| ***Ctnnd1*** | Catenin delta 1 | Cadherin associated protein | GC11P058206 |
| ***Gja1*** | Gap junction protein alpha 1 | Gap junction | GC06P121436 |
| ***Gja4*** | Gap junction protein alpha 4 | Gap junction | GC01P034792 |
| ***Jup*** | Junction plakoglobulin | Desmosome maintenance | GC17M041754 |
| ***Ocln*** | Occludin | Positive regulation | GC05P069492 |
| ***Tjp2*** | Tight junction protein 2 | Positive regulation | GC09P069121 |

* very low expression in non-neuronal cell types (database: MGI.org and genecards.org)

**Table S2: Cell types expressing genes modified by 2g exposure**

| Effect of 2g | Genes | Relative expressions in non-neuronal cell types |
|---|---|---|
| ↑ | ***Gja4*** | PC=SMC>EC>FB>MG>>OL-ACØ |
| ↑ | ***Ctnnd1*** | EC>PC-SMC>FB-OL-AC>MG |
| ↑ | ***Actn1*** | SMC>PC-EC-FB>MG-OL-AC |
| ↓ | ***Cdh2*** | PC-AC>SMC>EC>OL-FB>MG |
| ↓ | ***Ocln*** | EC>>>PC-SMC>AC>FB-OL>MGØ |
| ↓ | ***Actn2*** | EC-PC>SMC-FB-MG-OL>ACØ |
| ↓ | ***Jup*** | EC-PC-OL>SMC-FB>AC-MG |
| ↓ | ***Actn4*** | EC-PC-SMC>OL-FB-AC>MG |
| ↓ | ***Tjp2*** | EC-AC-OL>PC-SMC>FB>MGØ |
| ↓ | ***Gja1*** | AC>>EC>FB>>PC>SMC>OL-MGØ |

AC: astrocyte; EC: endothelial cell; FB:fibroblast; MG: microglia; OL: oligodendrocyte, PC: pericyte; SMC: smooth muscle cell (database: betsholtzlab.org).↑ upregulated, ↓ downregulated.

**Table S3: Comparison of gene expressions with database from Pulga et al., 2016**

| Gene name | 2g, 24h | 3g, 21 days [1] | Placebo [1] | Acute stress [1] | Chronic stress [1] |
|---|---|---|---|---|---|
| ***Gja4*** | ↑ | Ø | Ø | Ø | Ø |
| ***Ctnnd1*** | ↑ | Ø | Ø | Ø | Ø |
| ***Actn1*** | ↑ | Ø | Ø | Ø | Ø |
| ***Cdh2*** | ↓ | Ø | Ø | Ø | Ø |
| ***Ocln*** | ↓ | Ø | Ø | Ø | Ø |
| ***Actn2*** | ↓ | Ø | Ø | Ø | Ø |
| ***Jup*** | ↓ | Ø | Ø | Ø | Ø |
| ***Actn4*** | ↓ | Ø | Ø | Ø | Ø |
| ***Tjp2*** | ↓ | Ø | Ø | Ø | Ø |
| *Gja1* | ↓ | Ø | Ø | Ø | Ø |
| *Gja5* | Ø | Ø | Ø | Ø | Ø |
| *Actb* | Ø | Ø | Ø | Ø | Ø |
| *Actg1* | Ø | Ø | Ø | Ø | Ø |
| *Cdh5* | Ø | Ø | Ø | Ø | Ø |
| *Cldn1* | Ø | ↑ | Ø | ↑ | Ø |
| *Cldn3* | Ø | Ø | Ø | Ø | Ø |
| *Cldn5* | Ø | Ø | ↓ | Ø | Ø |
| *Ctnna1* | Ø | Ø | Ø | Ø | Ø |
| *Ctnnb1* | Ø | Ø | Ø | Ø | Ø |
| *Dsp* | Ø | Ø | Ø | Ø | Ø |
| *F11r* | Ø | Ø | Ø | Ø | Ø |
| *Jam2* | Ø | Ø | Ø | Ø | Ø |
| *Tjp1* | Ø | Ø | Ø | Ø | Ø |
| *Vim* | Ø | Ø | Ø | Ø | Ø |

↑ upregulated, ↓ downregulated, Ø not changed.
